# Profile and context of the work of physicians in the More Doctors for Brazil Project: a cross-sectional study, Brazil, 2024

**DOI:** 10.1590/S2237-96222026v35e20251297.en

**Published:** 2026-08-23

**Authors:** Rhaine Borges Santos Pedreira, Elzo Pereira Pinto, Marina Luna Pamponet, Ana Luiza Queiroz Vilasbôas, Silvana Lima Vieira, Elvira Caires de Lima, Nília Maria de Brito Lima Prado, Rosana Aquino

**Affiliations:** 1 Universidade Federal da Bahia, Instituto de Saúde Coletiva, Salvador, BA, Brazil Universidade Federal da Bahia Instituto de Saúde Coletiva Salvador BA Brazil; 2 Instituto Gonçalo Moniz, Centro de Integração de Dados e Conhecimentos para Saúde, Salvador, BA, Brazil Instituto Gonçalo Moniz Centro de Integração de Dados e Conhecimentos para Saúde Salvador BA Brazil; 3 Universidade do Estado da Bahia, Salvador, BA, Brazil Universidade do Estado da Bahia Salvador BA Brazil; 4 Universidade Federal da Bahia, Instituto Multidisciplinar em Saúde, Vitória da Conquista, BA, Brazil Universidade Federal da Bahia, Instituto Multidisciplinar em Saúde Vitória da Conquista BA Brazil

**Keywords:** Health Workforce, Health Consortia, Physician Distribution, Brazilian Unified Health System, Cross-Sectional Studies, Fuerza Laboral en Salud, Consorcios de Salud, Distribución de Médicos, Sistema Único de Salud, Estudios Transversales

## Abstract

**Objective:**

To describe the profile of physicians working in the More Doctors for Brazil (*Mais Médicos para o Brasil*) Project in 2024 and to analyze association between being an exchange physician and individual and contextual characteristics.

**Methods:**

This was a cross-sectional study with administrative data obtained from the program. The outcome was being an exchange physician, identified by type of professional registration (exchange physician or being registered with a Regional Medical Council in Brazil). Independent variables included individual characteristics of assigned health facilities and municipalities. Descriptive, bivariate and multivariate analyses were performed, with estimation of prevalence rates, prevalence ratios (PR), and 95% confidence intervals (95%CI).

**Results:**

Data on 19,418 physicians were analyzed. We found a predominance of female physicians, of White or Asian race/color, aged 30-49 years, and who were born in Brazil. Exchange physician prevalence was higher among male physicians (PR 1.10; 95%CI 1.07; 1.14), those aged 30-49 years (PR 1.22; 95%CI 1.17; 1.28) or ≥50 years (PR 1.18; 95%CI 1.12; 1.25), foreigners (PR 1.33; 95%CI 1.28; 1.39), and those working in establishments serving *quilombola* populations (PR 1.11; 95%CI 1.04; 1.18) and riverside populations (PR 1.35; 95%CI 1.24; 1.46). Higher exchange physician prevalence rates occurred in the Southeast, North, South and Midwest regions of Brazil, and in remote and adjacent municipalities.

**Conclusion:**

The study showed that the program's workforce is predominantly Brazilian, with a high percentage of exchange physicians. The participation of physicians with temporary and exclusive registration in the program highlights the importance of training, continuing education and supervision initiatives in primary health care.

## Resumen

**Objetivo:**

Describir el perfil de los médicos trabajando en el Proyecto *Mais Médicos para o Brasil* en 2024 y analizar la asociación entre ser médico de intercambio y las características individuales y contextuales.

**Métodos:**

Estudio transversal con datos administrativos del programa. La variable de resultado fue ser médico de intercambio, identificado por el tipo de registro profesional (médico de intercambio o registro con un Consejo Médico Regional de Brasil). Las variables independientes incluyeron características individuales de los centros de salud y municipios de asignación. Se realizaron análisis descriptivos, bivariados y multivariados, con estimación de prevalencias, razones de prevalencia (RP) e intervalos de confianza del 95% (IC95%).

**Resultados:**

Se analizaron datos de 19.418 médicos. Se observó un predominio de mujeres, de raza/color blanca o amarilla, con edades comprendidas entre 30 y 49 años y nacidas en Brasil. La prevalencia de médicos de intercambio fue mayor entre los profesionales varones (PR 1,10; IC95%: 1,07; 1,14), los de 30 a 49 años (PR 1,22; IC95%: 1,17; 1,28) o mayores de 50 años (PR 1,18; IC95%: 1,12; 1,25), los extranjeros (PR 1,33; IC95: 1,28; 1,39) y los que trabajaban en establecimientos que atendían a poblaciones *quilombolas* (PR 1,11; IC95%: 1,04; 1,18) y ribereñas (PR 1,35; IC95%: 1,24; 1,46). Las mayores prevalencias de médicos de intercambio se registraron en las regiones Sudeste, Norte, Sur y Centro-Oeste, así como en municipios remotos y adyacentes.

**Conclusión:**

El estudio demostró que el personal del programa es predominantemente brasileño, con un alto porcentaje de médicos de intercambio. La participación de médicos con inscripciones temporales y exclusivas en el programa refuerza la importancia de las iniciativas de capacitación, formación continua y supervisión en la atención primaria de salud.

**Aspectos éticos**

This research respected ethical principles, having obtained the following approval data: Research ethics committee: Universidade Federal da Bahia;

Opinion number: 6,152,744;

Approval date: 29/6/2023;

Certificate of submission for ethical appraisal: 70709523.8.0000.5030. Research ethics committee: Pan American Health Organization;

Opinion number: PAHOERC 0688.02;

Approval date: 11/8/2023;

Certificate of submission for ethical appraisal: Not applicable. Informed consent record: Waived.

## Introduction

Shortage and poor distribution of doctors pose a challenge to health systems in several countries^1^. In Brazil, this scenario compromises equity in access to care, especially in more remote and vulnerable regions, and requires adoption of public policies aimed at providing and retaining these professionals^2^.

The More Doctors Program (*Programa Mais Médicos*, PMM), established in 2013^3^, has become one of the main public policies aimed at overcoming the shortage and poor distribution of doctors in primary health care in Brazil. The Program was based on three complementary pillars: expanding and reformulation of medical training in terms of undergraduate and residency programs; emergency provision of doctors through the More Doctors for Brazil Project (*Projeto Mais Médicos para o Brasil*, PMMB); and the improvement of health center infrastructure^4^. This measure was essential in order to expand the presence of doctors in historically underserved regions, such as rural and remote areas, which promoted significant impacts on expanding primary health care coverage and guaranteeing access to health^5^^,^^6^.

Despite its relevance, the PMM became weakened between 2018 and 2022, a period in which the then-elected president of Brazil questioned the quality of the work of Cuban doctors, which resulted in the end of cooperation with Cuba. Consequently, most of these doctors left Brazil, intensifying the shortage of doctors in historically underserved regions^6^. In an attempt to reposition the policy, the federal government at the time launched the Doctors for Brazil Program (*Programa Médicos pelo Brasil*, PMpB) in 2019, focusing on the provision of doctors^7^. However, due to the slow implementation and limited reach of the PMpB, as well as changes in the Brazilian political context, the PMMB was relaunched in 2023, with emphasis on rebuilding health teams, professional training and retaining doctors in priority areas^8^.

International studies have shown that the workforce composition of physician provision programs is predominantly male^9^, with a significant participation of foreigners or graduates from countries other than those where they were born^4^^,^^10^. Evidence has also pointed to a higher frequency of foreign doctors working in challenging professional contexts, making it essential to have these professionals in order to mitigate disparities in medical care^11^.

Regarding the PMMB, the program is composed of physicians with different educational backgrounds and types of professional registration, resulting in a heterogeneous workforce. Among physicians with professional registration in Brazil, there are Brazilians and foreigners, trained in Brazil or with validated diplomas. Exchange physicians, on the other hand, are graduates from abroad, whether foreigners or Brazilians^4^. These professionals receive provisional professional registration granted by the Ministry of Health, with qualification restricted to the scope of the PMMB, and are required to participate in the Reception and Evaluation Module, aimed at professional qualification, integration into the guidelines of the Brazilian Unified Health System (*Sistema Único de Saúde*, SUS) and the local sociocultural context^12^^,^^13^.

Differences in training, prior professional experience, institution and working conditions can influence both the process of adaptation to the health system and professional performance, as suggested by a study on physician provision in remote and vulnerable areas^9^. However, evidence on how these differences are distributed among PMMB professionals and whether they are associated with sociodemographic and contextual characteristics is still limited. In this sense, the distinction regarding type of professional registration is a relevant analytical dimension for understanding the composition and challenges of the program's medical workforce, especially in territories that are vulnerable both in social and health terms.

Considering the strategic role of the PMMB in addressing inequalities in access to healthcare, understanding the composition of this workforce is fundamental to supporting the planning of public policies, identifying weaknesses in policy implementation and improving strategies for provision, distribution and retention of doctors in the SUS, especially in priority regions.

The objective of this study was to describe the profile of physicians working in the More Doctors for Brazil Project in 2024 and to analyze association between being an exchange physician and individual and contextual characteristics, based on the premise that type of professional registration is a marker of training trajectories and conditions of professional inclusion in the program.

## Methods 

Design and setting

This is a cross-sectional study developed within the context of the PMMB, a Brazilian Ministry of Health policy aimed at providing doctors and reducing inequalities in the distribution of these professionals in primary health care, especially in regions where there is shortage of care.

Participants, sample and eligibility criteria

The study population consisted of all physicians working in the PMMB in Brazil in March 2024. These professionals joined the program through calls for applications published between 2017 and 2023, corresponding to cycles 15-36.

Of the total of 19,859 physicians, 441 professionals allocated to Special Indigenous Health Districts were excluded, since these territories are not linked to a single municipality, thus making the contextual characteristic indicators selected for this study unavailable. The final sample consisted of 19,418 physicians working in Brazilian municipalities.

Data source and variables 

The data used in the study came from the Program Management System and were provided by the Ministry of Health. The database contained individual information on physicians working in the PMMB and was supplemented with variables relating to municipality profile and the health service network of the municipalities where they worked, obtained from publicly available databases of the Brazilian Institute of Geography and Statistics and the National Health Establishment Registry (*Cadastro Nacional de Estabelecimentos de Saúde*).

The social vulnerability index, provided by the Institute for Applied Economic Research (*Instituto de Pesquisa Econômica Aplicada*)^14^, was also incorporated. Database integration was performed by means of deterministic data matching, using the municipality code for linking contextual information and the health facility code for retrieving the characteristics of the units where the physicians worked.

The National Health Establishment Registry is the national database responsible for registering and standardizing information about health establishments in Brazil, gathering data on location, type of unit, services provided, human resources, infrastructure and equipment, and covering both public and private institutions. Each establishment has a unique code, which enables its integration with other information systems^15^.

In this study, the dependent variable was being an exchange physician (yes/no), derived via identification of type of professional registration (exchange physician versus CRM Brazil), held on the Ministry of Health Program Management System. The event was defined as the status of being an exchange physician, while the reference category was physicians registered with a Brazilian Regional Council of Medicine (*Conselho Regional de Medicina*) (CRM Brazil).

The independent variables were divided into three blocks:

Individual characteristics: sex (female or male), age group in years (22-29, 30-49 or ≥50) and country of birth (Brazil or other). The race/color variable was categorized into: White or Asian, Black or mixed-race, and Indigenous, whereby the White and Asian categories were grouped together due to the relatively low frequency of the latter and in order to ensure stability of the estimates. We recognize, however, that this procedure may limit the distinction between these categories in the analysis;

Characteristics of the establishments where the doctors worked and characteristics of the health teams: health establishment with internet connection (yes/no), establishments serving the *quilombola* population (yes/no), and establishments serving the riverside population (yes/no). Existence of a minimum set of physical facilities for health care (yes/no) was also included in the set of variables - considering clinical facilities, immunization room, dental treatment room and dressing room;

Contextual characteristics, referring to the municipalities where these professionals worked: geographic region (North, Northeast, Southeast, South and Midwest) and existence of hospital beds in the municipality (yes/no). The following categories were considered regarding rural/urban typology: urban; adjacent (including intermediate adjacent and rural adjacent); and remote (including intermediate remote and rural remote)^16^. The social vulnerability index (very low/low vulnerability, medium vulnerability, and high/very high vulnerability), estimated by the Institute for Applied Economic Research^14^, was also used. 

Bias

As this study used secondary administrative data, biases related to record quality may have occurred. Part of the information used was obtained from the National Health Establishment Registry, which, being an administrative database, may present inconsistencies or outdated records. In addition, the study used data from the Ministry of Health Project Management System regarding the hiring and allocation of physicians participating in the PMMB, the administrative nature of which suggests greater control over the quality of this information. In order to reduce possible inconsistencies in the records used, consistency checks were performed on the databases.

Statistical methods

Descriptive analysis of the data was performed, with calculation of absolute and relative frequencies, presented in tables. 

After applying Pearson's chi-square test, variables with a p-value<0.05 were used in unadjusted and adjusted logistic regression models, with estimation of prevalence ratios (PR) and their 95% confidence intervals (95%CI). In the regression models, odds ratios (OR) were transformed into PR, as per the methodology proposed^17^.

The statistical analyses were performed using Stata version 17.0.

## Results 

Among the 19,418 physicians active in the PMMB in March 2024 working in Brazilian municipalities, the majority were female (54.0%), aged between 30-49 years (69.5%), of White or Asian race/color (64.3%), and born in Brazil (85.6%). Most worked in establishments with a minimum set of physical facilities (58.2%) and internet access (78.4%). A higher concentration was observed in the Southeast (31.9%) and Northeast (30.3%) regions, in urban municipalities (63.0%), and which had hospital beds (89.5%). 32.1% were in contexts of high or very high social vulnerability. Work in units serving *quilombola* (6.9%) or riverside (3.7%) populations was less frequent. Regarding type of professional registration, 8,024 (41.3%) were classified as exchange physicians (Table 1).

In the bivariate analysis, prevalence of exchange physicians was higher among males (43.9%), Indigenous physicians (52.5%), those ≥50 years old (45.7%), and those born abroad (56.5%). It was also higher in establishments without a minimum set of physical facilities (43.3%) and in those serving *quilombola* (45.6%) or riverside (70.5%) populations. At the municipal level, higher prevalence of exchange physicians was observed in localities without hospital beds (49.4%), in the North (58.1%), Southeast (48.7%), and Southern (48.4%) regions, and especially in remote municipalities (76.6%). No statistically significant differences were observed according to type of professional registration in relation to internet availability at the establishment (p-value 0.419) or the social vulnerability index (p-value 0.287) (Table 2).

In multivariate analysis, prevalence of exchange physicians remained higher among males (PR 1.10; 95%CI 1.07; 1.14), those aged 30-49 years (PR 1.22; 95%CI 1.17; 1.28), and those ≥50 years or older (PR 1.18; 95%CI 1.12; 1.25), as well as among foreign physicians (PR 1.33; 95%CI 1.28; 1.39). No association was observed between race/color and type of professional registration (Table 3).

Regarding the work context, there was a higher prevalence of exchange physicians in units that serve *quilombola* populations (PR 1.11; 95%CI 1.04; 1.18) and riverside populations (PR 1.35; 95%CI 1.24; 1.46). Compared to the Northeast, prevalence was higher in the Southeast (PR 1.91; 95%CI 1.85; 1.98), North (PR 1.78; 95%CI 1.72; 1.85), South (PR 1.72; 95%CI 1.66; 1.78), and Midwest (PR 1.27; 95%CI 1.20; 1.35) regions. Remote municipalities showed almost double the prevalence of exchange physicians (PR 1.94; 95%CI 1.87; 2.02), and adjacent municipalities showed 33.0% higher prevalence of exchange physicians (PR 1.33; 95%CI 1.29; 1.38) compared to urban municipalities. Existence of hospital beds in the municipality and presence of a minimum set of physical facilities in the health establishment were not associated with being an exchange physician in the final model (Table 3).

**Table 1:** Individual characteristics and characteristics of the municipalities in which More Doctors for Brazil Project (PMMB) physicians were working. Brazil, March 2024 (n=19,418)

Variables	n (%)
Individual characteristics	
Sex	
Female	10,496 (54.0)
Male	8,922 (46.0)
Race/color	
White or Asian	12,489 (64.3)
Black or mixed-race	6,865 (35.4)
Indigenous	61 (0.3)
Age group (years)	
22-29	3,863 (19.9)
30-49	13,503 (69.5)
≥50	2,052 (10.6)
Country of birth	
Brazil	16,617 (85.6)
Other	2,801 (14.4)
Type of professional registration	
CRM Brazil^a^	11,394 (58.7)
Exchange physician	8,024 (41.3)
Health establishment and health team characteristics	
Minimum set of physical facilities for health care^b^	
No	8,115 (41.8)
Yes	11,303 (58.2)
Health establishment with internet connection	
No	4,062 (21.6)
Yes	14,776 (78.4)
Establishments serving the quilombola population	
No	17,495 (93.1)
Yes	1,292 (6.9)
Establishments serving the riverside population	
No	18,102 (96.3)
Yes	685 (3.7)
Municipality contextual characteristics	
Existence of hospital beds in the municipality	
No	2,038 (10.5)
Yes	17,380 (89.5)
Geographic region	
Northeast	5,909 (30.3)
Midwest	1,218 (6.3)
South	3,077 (15.9)
Southeast	6,193 (31.9)
North	3,021 (15.6)
Rural/urban typology	
Urban	12,239 (63.0)
Adjacent	6,044 (31.1)
Remote	1,135 (5.9)
Social vulnerability index	
Very low/low vulnerability	7,758 (39.9)
Medium vulnerability	5,434 (28.0)
High/very high vulnerability	6,226 (32.1)

a Registered with a Brazilian Regional Medical Council (CRM Brazil); ^b^Existence of clinical facilities, immunization room, dental treatment room and dressing room.

**Table 2:** Prevalence of More Doctors for Brazil Project exchange physicians, according to individual, health establishment and municipality characteristics. Brazil, March 2024 (n=19,418)

Variables	CRM Brazil^a^	Exchange physician	p-value
	n (%)	n (%)	
Individual characteristics			
Sex			<0.001
Female	6,388 (60.9)	4,108 (39.1)	
Male	5,006 (56.1)	3,916 (43.9)	
Race/color			0.023
White or Asian	7,263 (58.2)	5,226 (41.8)	
Black or mixed-race	4,099 (59.7)	2,766 (40.3)	
Indigenous	29 (47.5)	32 (52.5)	
Age group (years)			<0.001
22-29	2,611 (67.6)	1,252 (32.4)	
30-49	7,669 (56.8)	5,834 (43.2)	
≥50	1,114 (54.3)	938 (45.7)	
Country of birth			<0.001
Brazil	10,176 (61.2)	6,441 (38.8)	
Other	1,218 (43.5)	1,583 (56.5)	
Health establishment and health team characteristics			
Minimum set of physical facilities for health care^b^			<0.001
No	4,603 (56.7)	3,512 (43.3)	
Yes	6,791 (60.1)	4,512 (39.9)	
Health establishment with internet connection			0.419
No	2,384 (58.7)	1,678 (41.3)	
Yes	8,776 (59.4)	6,000 (40.6)	
**Establishments serving the *quilombola* population**			<0.001
No	10,433 (59.6)	7,062 (40.4)	
Yes	703 (54.4)	589 (45.6)	
Establishments serving the riverside population			<0.001
No	10,934 (60.4)	7,168 (39.6)	
Yes	202 (29.5)	483 (70.5)	
Municipality contextual characteristics			
Existence of hospital beds in the municipality			<0.001
No	1,032 (50.6)	1,006 (49.4)	
Yes	10,362 (59.6)	7,018 (40.4)	
Geographic region			<0.001
Northeast	4,526 (76.6)	1,383 (23.4)	
Midwest	832 (68.3)	386 (31.7)	
South	1,589 (51.6)	1,488 (48.4)	
Southeast	3,180 (51.3)	3,013 (48.7)	
North	1,267 (41.9)	1,754 (58.1)	
Rural/urban typology			<0.001
Urban	7,636 (62.4)	4,603 (37.6)	
Adjacent	3,492 (57.8)	2,552 (42.2)	
Remote	266 (23.4)	869 (76.6)	
Social vulnerability index			0.287
Very low/low vulnerability	4,521 (58.3)	3,237 (41.7)	
Medium vulnerability	3,237 (59.6)	2,197 (40.4)	
High/very high vulnerability	3,636 (58.4)	2,590 (41.6)	

a Registered with a Brazilian Regional Medical Council (CRM Brazil); ^b^Existence of clinical facilities, immunization room, dental treatment room and dressing room.

**Table 3:** Estimated prevalence ratios (PR) and 95% confidence intervals (95%CI) for association between More Doctors for Brazil Project (PMMB) exchange physicians and individual and contextual characteristics. Brazil, March 2024 (n=19,418)

Variables	Unadjusted model	Adjusted model
	PR (95%CI)	PR (95%CI)
Individual characteristics		
Sex		
Female	1.00	1.00
Male	1.12 (1.08; 1.16)	1.10 (1.07; 1.14)
Race/color		
White or Asian	1.00	1.00
Black or mixed-race	0.96 (0.93; 1.00)	1.00 (0.97; 1.03)
Indigenous	1.26 (0.99; 1.60)	1.10 (0.83; 1.44)
Age group (years)		
22-29	1.00	1.00
30-49	1.32 (1.26; 1.38)	1.22 (1.17; 1.28)
≥50	1.35 (1.28; 1.42)	1.18 (1.12; 1.25)
Country of birth		
Brazil	1.00	1.00
Other	1.46 (1.40; 1.51)	1.33 (1.28; 1.39)
Health establishment and health team characteristics		
Minimum set of physical facilities for health care^a^		
No	1.00	1.00
Yes	0.92 (0.89; 0.95)	1.00 (0.97; 1.04)
**Establishments serving the *quilombola* population**		
No	1.00	1.00
Yes	1.13 (1.06; 1.20)	1.11 (1.04; 1.18)
Establishments serving the riverside population		
No	1.00	1.00
Yes	1.78 (1.68; 1.87)	1.35 (1.24; 1.46)
Municipality contextual characteristics		
Existence of hospital beds in the municipality		
No	1.00	1.00
Yes	0.82 (0.78; 0.86)	0.99 (0.94; 1.05)
Geographic region		
Northeast	1.00	1.00
Midwest	1.23 (1.16; 1.31)	1.27 (1.20; 1.35)
South	1.67 (1.61; 1.73)	1.72 (1.66; 1.78)
Southeast	1.76 (1.70; 1.82)	1.91 (1.85; 1.98)
North	1.94 (1.88; 2.00)	1.78 (1.72; 1.85)
Rural/urban typology		
Urban	1.00	1.00
Adjacent	1.11 (1.08; 1.15)	1.33 (1.29; 1.38)
Remote	1.99 (1.92; 2.06)	1.94 (1.87; 2.02)

a Existence of clinical facilities, immunization room, dental treatment room and dressing room.

## Discussion 

The results of this study indicated that the PMMB is predominantly composed of doctors born in Brazil. Even so, a significant participation of exchange doctors was observed, especially in contexts of greater vulnerability and in remote territories. It was also observed that these professionals worked in establishments with minimum operating and internet connectivity conditions, with more frequent concentration in services that serve historically underserved populations, such as *quilombola* and riverside communities, as well as in hard-to-access areas. The findings also highlighted the persistent dependence on professionals trained abroad to guarantee healthcare in these territories.

Standing out among the limitations of this study is the exclusion of physicians working in Special Indigenous Health Districts. Although they represented a small portion of the professionals working in the PMMB in March 2024, they performed their activities in territories marked by significant challenges related to the provision and retention of physicians. Furthermore, this study only included physicians belonging to the PMMB, excluding those working in primary health care services under the rules of the BMpB, created in 2019. These are programs with distinct regulations, employment modalities and provision mechanisms, as well as having their own databases and administrative flows. Joint inclusion of these professionals in the analyses could lead to inappropriate comparisons, which is why the study focused on PMMB physicians. The scope of the conclusions, therefore, was restricted to just one of the provision programs in effect during the period. Finally, this was a cross-sectional study, which allows for identification of association, but not for establishment of causal relationships, and is therefore subject to possible biases inherent in the use of secondary data.

The predominance of females in the PMMB workforce reflects the trend of feminization in medicine, already described in Brazilian medical demography^18^. Considerable participation of Brazilian doctors under 30 years of age was also observed, indicating the program's role as a space for professional inclusion as well as following the trend of rejuvenation of the category in Brazil^18^. Despite syntheses of evidence on the PMMB^8^^,^^19^^,^^20^, studies specifically describing the sociodemographic profile of these professionals, at the national or regional level, are still scarce, limiting comparisons. Research conducted in the state of Paraíba with doctors linked to the program between 2015 and 2016 identified a predominance of professionals with up to five years of work experience since graduation^21^.

This study pointed to a change in relation to the country of birth of the professionals participating in the PMMB, when compared to the first cycles between 2013-2014. In 2024, more than 80.0% of the doctors working in the program were Brazilian, while in July 2014, 79.0% were Cuban and were recruited and allocated to primary health care services by means of international cooperation with the Cuban government and the Pan American Health Organization^22^.

The increase in the proportion of Brazilian doctors, especially those trained in Brazil, in the composition of the PMMB workforce can be attributed, in part, to the expansion of medical courses in recent decades and especially after the creation of the PMM in 2013. Evidence showed that between January 2014 and February 2022, 135 new medical courses were opened, 68 of which were as a result of the PMM, with the majority being private courses, especially in the Northeast, Southeast and Southern regions, and located in areas with greater availability of doctors^23^. However, when considering only the implementation of new medical courses in Brazilian federal universities, the creation of 30 new courses was documented between 2013 and 2015, all of them located in municipalities outside the major urban centers and capitals, distributed across the five Brazilian macro-regions^24^. Furthermore, the recent expansion of medical residency positions, especially in Northeastern states, also signals a process of medical training that is both decentralized and located in inner-state regions, potentially associated with greater retention of professionals in the regions where they specialize^2^.

Despite the increase in Brazilian doctors in the PMMB, the high proportion of exchange doctors still poses challenges to professional retention and building lasting employment relationships in primary health care. The work of doctors trained abroad with temporary and exclusive registration with the program is more susceptible to variations in the political agenda, as occurred in 2018, with the departure of Cuban doctors after the election of former President Jair Bolsonaro^25^. Abrupt discontinuation of these professionals can compromise continuity of care and quality of health services, as it is associated reduction in the supply of primary health care services and increased use of urgent and emergency services primary health care and greater recourse to specialized and emergency services, with negative impacts on health outcomes^26^.

Exchange physicians, predominantly male and in older age groups, were mainly located in territories with historically challenging conditions for the provision and retention of physicians, and in remote areas. These findings reinforced that, despite advances in the provision of physicians in primary health care since the creation of the PMM in 2013, allocation of professionals trained in Brazil to more vulnerable territories remains challenging. Strategies to attract physicians trained abroad, both Brazilian and foreign, remain relevant for ensuring their presence in primary care in Brazil.

The results also showed higher frequency of exchange doctors in establishments serving *quilombola* and riverside populations. This finding corroborated literature that pointed to the PMMB as an effective policy for providing care to historically underserved populations, expanding access to primary care in vulnerable communities^5^. Evidence indicated that foreign doctors, especially Cubans, showed greater adaptation to these contexts due to their training oriented towards prevention, humanization of care, and experience in international missions, which favors medical practice aligned with the needs of remote areas^27^^,^^28^. This greater presence should also be analyzed, however, in light of structural factors associated with the low retention of Brazilian doctors in historically underserved areas, such as less attractive remuneration, precarious working conditions, and socialization difficulties^4^. Allocation of these professionals in hard-to-reach territories reinforces the program's role in reducing social and territorial health inequalities and highlights the limitations of the domestic market in meeting the needs of these areas without the contribution of exchange doctors.

An aspect worthy of highlight in this study is the new pattern observed among exchange physicians, who were predominantly Brazilian. This finding relativized the immediate association between type of registration and country of birth, revealing that the PMMB brings together diverse educational trajectories. Many Brazilians sought undergraduate studies in other countries due to the high competitiveness for admission to medical courses in Brazil, especially before the recent expansion of national medical schools^18^. Upon returning, these professionals find in the PMMB a legal way of starting work in the SUS, within the scope of primary health care services, without facing the regulatory requirements related to diploma revalidation^3^^,^^4^.

In this context, the importance of the Reception and Evaluation Module, mandatory for exchange doctors, was highlighted. The module seeks to ensure minimum qualification for working in the SUS, by introducing these professionals to primary health care guidelines, Brazil's epidemiological specificities and the sociocultural particularities of the communities where they will work. For Brazilians trained abroad, the Reception and Evaluation Module represents not only a formal requirement, but also an opportunity for updating and aligning with practice in the SUS. The module thus fulfills a dual function: it responds to criticisms about the inclusion of professionals trained outside Brazil without immediate proof of competence and acts as a pedagogical device for integration and regulation, which contributes to the effectiveness of the program in territories of greater social and health vulnerability^13^.

It is also interesting to note that higher prevalence of exchange doctors was observed in all Brazilian regions, except for the Northeast region. Although this region contains many municipalities in vulnerable situations, the expansion and decentralization of medical education, especially in federal universities, may explain the lower dependence on doctors trained outside Brazil^24^. Furthermore, approximately 50.0% of all doctors in the PMpB are concentrated in the Northeast region, which may explain the lower dependence on exchange doctors for filling vacancies in primary health care services^29^.

This study was one of the first nationwide studies to describe the sociodemographic profile and factors associated with the type of professional registration of physicians working in the PMMB, and it showed that the program's workforce is predominantly composed of Brazilian physicians, although there is considerable participation of exchange physicians, especially in areas that are vulnerable both in social and health terms.

The association between type of professional registration, individual characteristics of physicians, and the structural and social conditions of the territories revealed distinct patterns of inclusion as primary health care workers. These findings highlighted the relevance of considering these dimensions in planning and evaluating physician provision policies, as well as the importance of training, supervision and continuing education initiatives to improve the performance of these professionals and promote their retention in contexts of greater vulnerability.

## Data Availability

The dataset that supports the results of this study is not publicly available.
