# Supplementary material for: Profile and context of the work of physicians in the More Doctors for Brazil Project: a cross-sectional study, Brazil, 2024
Source: Epidemiol Serv Saude. 2026 Aug 23;35:e20251297. doi: 10.1590/S2237-96222026v35e20251297.en (PMC13686426; doi:10.1590/S2237-96222026v35e20251297.en)
Supplement: Translation

# Translation: Perfil e contexto de atuação dos médicos do Projeto Mais Médicos para o Brasil: estudo transversal, Brasil, 2024

**DOI:** 10.1590/S2237-96222026v35e20251297.pt

**Authors:** Rhaine Borges Santos Pedreira, Elzo Pereira Pinto, Marina Luna Pamponet, Ana Luiza Queiroz Vilasbôas, Silvana Lima Vieira, Elvira Caires de Lima, Nília Maria de Brito Lima Prado, Rosana Aquino

**Affiliations:** 1 Universidade Federal da Bahia, Instituto de Saúde Coletiva, Salvador, BA, Brasil Universidade Federal da Bahia Instituto de Saúde Coletiva Salvador BA Brasil; 2 Instituto Gonçalo Moniz, Centro de Integração de Dados e Conhecimentos para Saúde, Salvador, BA, Brasil Instituto Gonçalo Moniz Centro de Integração de Dados e Conhecimentos para Saúde Salvador BA Brasil; 3 Universidade do Estado da Bahia, Salvador, BA, Brasil Universidade do Estado da Bahia Salvador BA Brasil; 4 Universidade Federal da Bahia, Instituto Multidisciplinar em Saúde, Vitória da Conquista, BA, Brasil Universidade Federal da Bahia, Instituto Multidisciplinar em Saúde Vitória da Conquista BA Brasil

**Keywords:** Mão de Obra em Saúde, Consórcios de Saúde, Distribuição de médicos, Sistema Único de Saúde, Estudos Transversais.

## Abstract

**Objetivo:**

Descrever o perfil dos médicos ativos no Projeto Mais Médicos para o Brasil em 2024 e analisar a associação entre ser médico intercambista e características individuais e contextuais.

**Métodos:**

Estudo transversal com dados administrativos do programa. O desfecho foi ser médico intercambista, identificado a partir do tipo de registro profissional (intercambista ou inscrição em Conselho Regional de Medicina no Brasil). As variáveis independentes incluíram características individuais dos estabelecimentos de saúde e municípios de alocação. Realizaram-se análises descritiva, bivariada e multivariada, com estimação de prevalências, razões de prevalência (RP) e intervalos de confiança de 95% (IC95%).

**Resultados:**

Foram analisados dados de 19.418 médicos. Observou-se predominância de profissionais do sexo feminino, de raça/cor branca ou amarela, com idade entre 30-49 anos e nascidos no Brasil. A prevalência de intercambistas foi maior entre profissionais do sexo masculino (RP 1,10; IC95% 1,07; 1,14), com 30-49 anos (RP 1,22; IC95% 1,17; 1,28) ou ≥50 anos (RP 1,18; IC95% 1,12; 1,25), estrangeiros (RP 1,33; IC95% 1,28; 1,39), alocados em estabelecimentos que atendem populações quilombolas (RP 1,11; IC95% 1,04; 1,18) e ribeirinhas (RP 1,35; IC95% 1,24; 1,46). Prevalências mais elevadas de intercambistas ocorreram nas regiões Sudeste, Norte, Sul e Centro-Oeste e em municípios remotos e adjacentes.

**Conclusão:**

O estudo evidenciou que a força de trabalho do programa é predominantemente brasileira, com elevado percentual de intercambistas. A atuação de médicos com registros temporários e exclusivos para o programa reforça a importância das iniciativas de treinamento, educação continuada e supervisão das práticas na atenção primária à saúde.

**Aspectos éticos**

Esta pesquisa respeitou os princípios éticos, obtendo os seguintes dados de aprovação: Comitê de ética em pesquisa: Universidade Federal da Bahia;

Número do parecer: 6.152.744;

Data de aprovação: 29/6/2023;

Certificado de apresentação de apreciação ética: 70709523.8.0000.5030. Comitê de ética em pesquisa: Organização Panamericana de Saúde;

Número do parecer: PAHOERC 0688.02;

Data de aprovação: 11/8/2023;

Certificado de apresentação de apreciação ética: Não se aplica. Registro do consentimento livre e esclarecido: Dispensado.

## Introdução 

A escassez e a má distribuição de médicos constituem um desafio para os sistemas de saúde em diversos países^1^. No Brasil, esse cenário compromete a equidade no acesso aos cuidados, especialmente em regiões mais remotas e vulneráveis, e exige a adoção de políticas públicas que visem o provimento e a fixação desses profissionais^2^.

O Programa Mais Médicos (PMM), instituído em 2013^3^, consolidou-se como uma das principais políticas públicas voltadas à superação da escassez e da má distribuição de médicos na atenção primária à saúde (APS) no Brasil. O programa foi fundamentado em três eixos complementares: a ampliação e a reformulação da formação médica quanto à graduação e às residências médicas; o provimento emergencial de médicos por meio do Projeto Mais Médicos para o Brasil (PMMB); e a qualificação da infraestrutura das unidades de saúde^4^. Essa medida foi essencial para ampliar a presença dos médicos em regiões historicamente desassistidas, como áreas rurais e remotas, o que promoveu impactos significativos na expansão da cobertura da APS e na garantia do acesso à saúde^5^^,^^6^.

Apesar da sua relevância, o PMM sofreu enfraquecimento entre 2018 e 2022, período em que o presidente eleito do Brasil à época questionou a qualidade do trabalho dos médicos cubanos, o que resultou no fim da cooperação com Cuba. Consequentemente, a maior parte desses profissionais deixou o país, o que intensificou a escassez de médicos em regiões historicamente desassistidas^6^. Para tentar reposicionar a política, o governo federal lançou, em 2019, o Programa Médicos pelo Brasil (PMpB), com foco no provimento de médicos^7^. Porém, devido à implementação lenta e ao alcance limitado do PMpB, além de mudanças no cenário político brasileiro, o PMMB foi relançado em 2023, com ênfase na recomposição das equipes, na formação profissional e na fixação de médicos em áreas prioritárias^8^.

Estudos internacionais apontaram que a composição da força de trabalho dos programas de provimento médico era majoritariamente masculina^9^, com expressiva participação de estrangeiros ou de graduados em países diferentes daqueles onde nasceram^4^^,^^10^. Evidências também apontaram para maior frequência de médicos estrangeiros com atuação em contextos profissionais desafiadores, de forma a ser imprescindível contar com esses profissionais para mitigar as disparidades na assistência médica^11^.

No que tange ao PMMB, o programa é composto por médicos com diferentes trajetórias formativas e tipos de registro profissional, o que resulta em uma força de trabalho heterogênea. Entre os médicos com registro profissional no Brasil, há brasileiros e estrangeiros, formados no país ou com diplomas validados. Já os médicos intercambistas são graduados no exterior, sejam estrangeiros ou brasileiros^4^. Esses profissionais recebem um registro profissional provisório concedido pelo Ministério da Saúde, com habilitação restrita ao âmbito do PMMB, e participam obrigatoriamente do Módulo de Acolhimento e Avaliação, voltado à qualificação profissional, à integração às diretrizes do Sistema Único de Saúde (SUS) e ao contexto sociocultural local^12^^,^^13^. 

Diferenças na formação, na experiência profissional prévia, na inserção institucional e nas condições de trabalho podem influenciar o processo de adaptação ao sistema de saúde e o desempenho profissional, conforme sugerido por estudo sobre provimento médico em áreas remotas e vulneráveis^9^. Ainda são limitadas, contudo, as evidências sobre como essas diferenças se distribuem entre os profissionais do PMMB e se estão associadas a características sociodemográficas e contextuais. Nesse sentido, a distinção quanto ao tipo de registro profissional constitui uma dimensão analítica relevante para compreender a composição e os desafios da força de trabalho médica do programa, especialmente em territórios social e sanitariamente vulneráveis.

Levando-se em consideração o papel estratégico do PMMB no enfrentamento das desigualdades no acesso à saúde, compreender a composição dessa força de trabalho é fundamental para subsidiar o planejamento de políticas públicas, identificar fragilidades na execução da política e aprimorar estratégias de provimento, distribuição e fixação de médicos no SUS, especialmente em regiões prioritárias.

Este estudo tem como objetivo descrever o perfil dos médicos ativos no Projeto Mais Médicos para o Brasil em 2024 e analisar a associação entre ser médico intercambista e características individuais e contextuais, com a premissa de que o tipo de registro constitui um marcador das trajetórias formativas e das condições de inserção profissional no programa.

## Métodos

Delineamento e contexto

Trata-se de um estudo transversal desenvolvido no contexto do PMMB, política do Ministério da Saúde voltada ao provimento de médicos e à redução das desigualdades na distribuição desses profissionais na APS, especialmente em regiões com escassez de assistência.

Participantes, amostra e critérios de elegibilidade

A população do estudo foi composta por todos os médicos ativos no PMMB no mês de março de 2024 no Brasil. Esses profissionais ingressaram no programa por meio de editais de convocação publicados entre 2017 e 2023, correspondentes aos ciclos 15º-36º.

Do total de 19.859 médicos, foram excluídos 441 profissionais alocados em Distritos Sanitários Especiais Indígenas, uma vez que esses territórios não estão vinculados a um único município, não sendo disponíveis os indicadores de características contextuais selecionados para este estudo. A amostra final foi composta por 19.418 médicos com atuação em municípios brasileiros.

Fonte de dados e variáveis

Os dados utilizados no estudo foram provenientes do Sistema de Gerenciamento de Programas e fornecidos pelo Ministério da Saúde. A base de dados continha informações individuais dos médicos ativos no PMMB e foi complementada com variáveis referentes ao perfil municipal e à rede de serviços de saúde do município de atuação, obtidas em bases públicas do Instituto Brasileiro de Geografia e Estatística e do Cadastro Nacional de Estabelecimentos de Saúde.

Também foi incorporado o índice de vulnerabilidade social, disponibilizado pelo Instituto de Pesquisa Econômica Aplicada^14^. A integração das bases de dados foi realizada através de pareamento determinístico dos dados, com utilização do código do município de atuação para a vinculação das informações contextuais e do código do estabelecimento de saúde para a recuperação das características da unidade onde o médico atuava.

O Cadastro Nacional de Estabelecimentos de Saúde é a base nacional responsável pelo registro e pela padronização das informações sobre os estabelecimentos de saúde no país, com dados sobre localização, tipo de unidade, serviços prestados, recursos humanos, infraestrutura e equipamentos e com abrangência de instituições públicas e privadas. Cada estabelecimento possui um código único, o que possibilita sua integração a outros sistemas de informação^15^.

Neste estudo, a variável dependente foi ser intercambista (sim/não), construída a partir da identificação do tipo de registro profissional (intercambista versus CRM Brasil), disponível no Sistema de Gerenciamento de Programas do Ministério da Saúde. Tratou-se como evento a condição de médico intercambista e como categoria de referência os médicos com inscrição no Conselho Regional de Medicina (CRM Brasil).

Foram considerados três blocos de variáveis independentes:

 Características individuais: sexo (feminino ou masculino), faixa etária em anos (22-29, 30-49 ou ≥50) e país de nascimento (Brasil ou outros). A variável raça/cor foi categorizada em: branca ou amarela, preta ou parda e indígena, com as categorias branca e amarela agrupadas devido à baixa frequência relativa desta última e para garantir estabilidade das estimativas. Reconhece-se, contudo, que esse procedimento pode limitar a distinção entre essas categorias na análise;

 Características dos estabelecimentos onde os médicos atuavam e das equipes de saúde: conexão com internet no estabelecimento de saúde (sim/não), estabelecimentos que atendem população quilombola (sim/não) e estabelecimentos que atendem população ribeirinha (sim/não). Também compôs o bloco de variáveis a existência de um conjunto mínimo de instalações físicas para assistência à saúde (sim/não) - com a consideração de instalações clínicas, sala de imunização, consultório odontológico e sala de curativo;

 Características contextuais, referentes aos municípios de atuação desses profissionais: região geográfica (Norte, Nordeste, Sudeste, Sul e Centro-Oeste) e existência de leitos hospitalares no município (sim/não). Para a tipologia rural/urbano foram consideradas as categorias: urbano; adjacente (com a contemplação de intermediário adjacente e rural adjacente); e remoto (com a contemplação de intermediário remoto e rural remoto)^16^. Utilizou-se ainda o índice de vulnerabilidade social (muito baixa/baixa vulnerabilidade, média vulnerabilidade e alta/muito alta vulnerabilidade), estimado pelo Instituto de Pesquisa Econômica Aplicada^14^.

Viés

Por se tratar de estudo com dados administrativos secundários, podem ocorrer vieses relacionados à qualidade do registro. Parte das informações utilizadas foi obtida do Cadastro Nacional de Estabelecimentos de Saúde (CNES), que, por se tratar de base administrativa, pode apresentar inconsistências ou desatualização de registros. Além disso, o estudo utilizou dados do Sistema de Gerenciamento de Projetos do Ministério da Saúde referentes à contratação e à alocação dos médicos participantes do PMMB, cuja natureza administrativa sugere maior controle da qualidade do preenchimento dessas informações. Para reduzir possíveis inconsistências nos registros utilizados, foram realizadas verificações de consistência nos bancos de dados.

Métodos estatísticos

Foi realizada uma análise descritiva dos dados, com cálculo de frequências absolutas e relativas, apresentadas em tabelas.

Após a aplicação do teste qui-quadrado de Pearson, as variáveis com p-valor<0,05 foram utilizadas em modelos bruto e ajustado de regressão logística, com estimação de razões de prevalência (RP) e seus intervalos de confiança de 95% (IC95%). Nos modelos de regressão, foi aplicada a transformação da razão de chance (*odds ratio*, OR) em RP, conforme metodologia proposta^17^.

As análises estatísticas foram realizadas no software Stata, versão 17.0.

## Resultados 

Entre os 19.418 médicos ativos no PMMB em março de 2024 com atuação em municípios brasileiros predominavam profissionais do sexo feminino (54,0%), com idade entre 30-49 anos (69,5%), de raça/cor branca ou amarela (64,3%) e nascidos no Brasil (85,6%). A maioria atuava em estabelecimentos com conjunto mínimo de instalações físicas (58,2%) e com conexão à internet (78,4%). Observou-se maior concentração nas regiões Sudeste (31,9%) e Nordeste (30,3%), em municípios urbanos (63,0%) e com leitos hospitalares (89,5%). 32,1% estavam em contextos de alta ou muito alta vulnerabilidade social. A atuação em unidades que atendem populações quilombolas (6,9%) ou ribeirinhas (3,7%) foi menos frequente. Quanto ao tipo de registro profissional, 8.024 (41,3%) eram classificados como intercambistas (Tabela 1). 

Na análise bivariada, a prevalência de intercambistas foi maior entre pessoas do sexo masculino (43,9%), indígenas (52,5%), com ≥50 anos (45,7%) e nascidas no exterior (56,5%). Também foi mais elevada em estabelecimentos sem conjunto mínimo de instalações físicas (43,3%) e naqueles que atendiam populações quilombolas (45,6%) ou ribeirinhas (70,5%). No âmbito municipal, observou-se maior prevalência de profissionais intercambistas em localidades sem leitos hospitalares (49,4%), nas regiões Norte (58,1%), Sudeste (48,7%) e Sul (48,4%) e, especialmente, em municípios remotos (76,6%). Não foram observadas diferenças estatisticamente significativas segundo o tipo de registro profissional em relação à disponibilidade de internet no estabelecimento (p-valor 0,419) nem ao índice de vulnerabilidade social (p-valor 0,287) (Tabela 2).

Na análise multivariada, a prevalência de intercambistas permaneceu maior entre pessoas do sexo masculino (RP 1,10; IC95% 1,07; 1,14), com 30-49 anos (RP 1,22; IC95% 1,17; 1,28) e com ≥50 anos ou mais (RP 1,18; IC95% 1,12; 1,25), bem como entre médicos estrangeiros (RP 1,33; IC95% 1,28; 1,39). Não se observou associação entre raça/cor e tipo de registro profissional (Tabela 3).

Quanto ao contexto de atuação, houve maior prevalência de intercambistas em unidades que atendem populações quilombolas (RP 1,11; IC95% 1,04; 1,18) e ribeirinhas (RP 1,35; IC95% 1,24; 1,46). Em comparação ao Nordeste, a prevalência foi mais elevada nas regiões Sudeste (RP 1,91; IC95% 1,85; 1,98), Norte (RP 1,78; IC95% 1,72; 1,85), Sul (RP 1,72; IC95% 1,66; 1,78) e Centro-Oeste (RP 1,27; IC95% 1,20; 1,35). Municípios remotos apresentaram quase o dobro da prevalência de intercambistas (RP 1,94; IC95% 1,87; 2,02), e municípios adjacentes apresentaram prevalência 33,0% maior de médicos intercambistas (RP 1,33; IC95% 1,29; 1,38) em relação aos urbanos. A existência de leitos hospitalares no município e a presença de conjunto mínimo de instalações físicas no estabelecimento não permaneceram associadas a ser intercambista no modelo final (Tabela 3).

**Tabela 1:** Características individuais e do município de atuação dos médicos ativos no Projeto Mais Médicos para o Brasil (PMMB). Brasil, março de 2024 (n=19.418)

Variáveis	n (%)
Características individuais	
Sexo	
Feminino	10.496 (54,0)
Masculino	8.922 (46,0)
Raça/cor	
Branca ou amarela	12.489 (64,3)
Preta ou parda	6.865 (35,4)
Indígena	61 (0,3)
Faixa etária (anos)	
22-29	3.863 (19,9)
30-49	13.503 (69,5)
≥50	2.052 (10,6)
País de nascimento	
Brasil	16.617 (85,6)
Outros	2.801 (14,4)
Tipo de registro profissional	
CRM Brasil^a^	11.394 (58,7)
Intercambista	8.024 (41,3)
Características dos estabelecimentos e equipes de saúde	
Conjunto mínimo de instalações físicas para assistência à saúde^b^	
Não	8.115 (41,8)
Sim	11.303 (58,2)
Conexão com internet no estabelecimento de saúde	
Não	4.062 (21,6)
Sim	14.776 (78,4)
Estabelecimentos que atendem população quilombola	
Não	17.495 (93,1)
Sim	1.292 (6,9)
Estabelecimentos que atendem população ribeirinha	
Não	18.102 (96,3)
Sim	685 (3,7)
Características contextuais dos municípios	
Existência de leitos hospitalares no município	
Não	2.038 (10,5)
Sim	17.380 (89,5)
Região geográfica	
Nordeste	5.909 (30,3)
Centro-Oeste	1.218 (6,3)
Sul	3.077 (15,9)
Sudeste	6.193 (31,9)
Norte	3.021 (15,6)
Tipologia rural/urbano	
Urbano	12.239 (63,0)
Adjacente	6.044 (31,1)
Remoto	1.135 (5,9)
Índice de vulnerabilidade social	
Muito baixa/baixa vulnerabilidade	7.758 (39,9)
Média vulnerabilidade	5.434 (28,0)
Alta/muito alta vulnerabilidade	6.226 (32,1)

a Inscrição no Conselho Regional de Medicina (CRM Brasil); ^b^Existência de instalações clínicas, sala de imunização, consultório odontológico e sala de curativo.

**Tabela 2:** Prevalência de médicos intercambistas do Projeto Mais Médicos para o Brasil, segundo características individuais, dos estabelecimentos e dos municípios de atuação. Brasil, março de 2024 (n=19.418)

Variáveis	CRM Brasil^a^	Intercambista	p-valor
	n (%)	n (%)	
Características individuais			
Sexo			<0,001
Feminino	6.388 (60,9)	4.108 (39,1)	
Masculino	5.006 (56,1)	3.916 (43,9)	
Raça/cor			0,023
Branca ou amarela	7.263 (58,2)	5.226 (41,8)	
Preta ou parda	4.099 (59,7)	2.766 (40,3)	
Indígena	29 (47,5)	32 (52,5)	
Faixa etária (anos)			<0,001
22-29	2.611 (67,6)	1.252 (32,4)	
30-49	7.669 (56,8)	5.834 (43,2)	
≥50	1.114 (54,3)	938 (45,7)	
País de nascimento			<0,001
Brasil	10.176 (61,2)	6.441 (38,8)	
Outros	1.218 (43,5)	1.583 (56,5)	
Características dos estabelecimentos e equipes de saúde			
Conjunto mínimo de instalações físicas para assistência à saúde^b^			<0,001
Não	4.603 (56,7)	3.512 (43,3)	
Sim	6.791 (60,1)	4.512 (39,9)	
Conexão com internet no estabelecimento de saúde			0,419
Não	2.384 (58,7)	1.678 (41,3)	
Sim	8.776 (59,4)	6.000 (40,6)	
Estabelecimentos que atendem população quilombola			<0,001
Não	10.433 (59,6)	7.062 (40,4)	
Sim	703 (54,4)	589 (45,6)	
Estabelecimentos que atendem população ribeirinha			<0,001
Não	10.934 (60,4)	7.168 (39,6)	
Sim	202 (29,5)	483 (70,5)	
Características contextuais dos municípios			
Existência de leitos hospitalares no município			<0,001
Não	1.032 (50,6)	1.006 (49,4)	
Sim	10.362 (59,6)	7.018 (40,4)	
Região geográfica			<0,001
Nordeste	4.526 (76,6)	1.383 (23,4)	
Centro-Oeste	832 (68,3)	386 (31,7)	
Sul	1.589 (51,6)	1.488 (48,4)	
Sudeste	3.180 (51,3)	3.013 (48,7)	
Norte	1.267 (41,9)	1.754 (58,1)	
Tipologia rural/urbano			<0,001
Urbano	7.636 (62,4)	4.603 (37,6)	
Adjacente	3.492 (57,8)	2.552 (42,2)	
Remoto	266 (23,4)	869 (76,6)	
Índice de vulnerabilidade social			0,287
Muito baixa/baixa vulnerabilidade	4.521 (58,3)	3.237 (41,7)	
Média vulnerabilidade	3.237 (59,6)	2.197 (40,4)	
Alta/muito alta vulnerabilidade	3.636 (58,4)	2.590 (41,6)	

a Inscrição no Conselho Regional de Medicina (CRM Brasil); ^b^Existência de instalações clínicas, sala de imunização, consultório odontológico e sala de curativo.

**Tabela 3:** Estimativas das razões de prevalência (RP) e dos intervalos de confiança (IC95%) para a associação entre ser intercambistas do Projeto Mais Médicos para o Brasil (PMMB) e as características individuais e contextuais. Brasil, março de 2024 (n=19.418)

Variáveis	Modelo bruto	Modelo ajustado
	RP (IC95%)	RP (IC95%)
Características individuais		
Sexo		
Feminino	1,00	1,00
Masculino	1,12 (1,08; 1,16)	1,10 (1,07; 1,14)
Raça/cor		
Branca ou amarela	1,00	1,00
Preta ou parda	0,96 (0,93; 1,00)	1,00 (0,97; 1,03)
Indígena	1,26 (0,99; 1,60)	1,10 (0,83; 1,44)
Faixa etária (anos)		
22-29	1,00	1,00
30-49	1,32 (1,26; 1,38)	1,22 (1,17; 1,28)
≥50	1,35 (1,28; 1,42)	1,18 (1,12; 1,25)
País de nascimento		
Brasil	1,00	1,00
Outros	1,46 (1,40; 1,51)	1,33 (1,28; 1,39)
Características dos estabelecimentos e equipes de saúde		
Conjunto mínimo de instalações físicas para assistência à saúde^a^		
Não	1,00	1,00
Sim	0,92 (0,89; 0,95)	1,00 (0,97; 1,04)
Estabelecimentos que atendem população quilombola		
Não	1,00	1,00
Sim	1,13 (1,06; 1,20)	1,11 (1,04; 1,18)
Estabelecimentos que atendem população ribeirinha		
Não	1,00	1,00
Sim	1,78 (1,68; 1,87)	1,35 (1,24; 1,46)
Características contextuais dos municípios		
Existência de leitos hospitalares no município		
Não	1,00	1,00
Sim	0,82 (0,78; 0,86)	0,99 (0,94; 1,05)
Região geográfica		
Nordeste	1,00	1,00
Centro-Oeste	1,23 (1,16; 1,31)	1,27 (1,20; 1,35)
Sul	1,67 (1,61; 1,73)	1,72 (1,66; 1,78)
Sudeste	1,76 (1,70; 1,82)	1,91 (1,85; 1,98)
Norte	1,94 (1,88; 2,00)	1,78 (1,72; 1,85)
Tipologia rural/urbano		
Urbano	1,00	1,00
Adjacente	1,11 (1,08; 1,15)	1,33 (1,29; 1,38)
Remoto	1,99 (1,92; 2,06)	1,94 (1,87; 2,02)

a Existência de instalações clínicas, sala de imunização, consultório odontológico e sala de curativo.

## Discussão

Os resultados deste estudo indicaram que o PMMB é composto predominantemente por médicos nascidos no Brasil. Ainda assim, observou-se participação relevante de médicos intercambistas, sobretudo em contextos de maior vulnerabilidade e em territórios remotos. Observou-se também que esses profissionais atuavam em estabelecimentos com condições mínimas de funcionamento e de conectividade, com concentração mais frequente em serviços que atendem populações historicamente desassistidas, como quilombolas e ribeirinhas, bem como em áreas de difícil provimento. Esses achados também evidenciaram a persistente dependência de profissionais formados no exterior para garantir a assistência nesses territórios.

Entre as limitações do estudo, destaca-se a exclusão dos médicos que atuavam em Distritos Sanitários Especiais Indígenas que, embora representassem pequena parcela dos profissionais ativos no PMMB em março de 2024, desempenhavam suas atividades em territórios marcados por grandes desafios relacionados ao provimento e à fixação de médicos. Além disso, este estudo contemplou apenas médicos vinculados ao PMMB, sem incluir aqueles que atuavam em serviços de APS sob as regras do PMpB, criado em 2019. Trata-se de programas com normativas, modalidades de vínculo e mecanismos de provimento distintos, além de bases de dados e fluxos administrativos próprios. A inclusão conjunta desses profissionais nas análises poderia gerar comparações inadequadas, razão pela qual o estudo concentrou-se nos médicos vinculados ao PMMB. O escopo das conclusões, então, restringiu-se a um dos programas de provimento vigentes no período. Por fim, tratou-se de estudo transversal, que permite identificar associações, mas não estabelecer relações de causalidade, com sujeição a possíveis vieses inerentes ao uso de dados secundários.

A predominância do sexo feminino na força de trabalho do PMMB reflete a tendência de feminização da medicina, já descrita na demografia médica brasileira^18^. Observou-se também participação expressiva de médicos brasileiros com menos de 30 anos, o que indicou o papel do programa como espaço de inserção profissional e de acompanhamento do rejuvenescimento da categoria no país^18^. Apesar da existência de sínteses de evidências sobre o PMMB^8^^,^^19^^,^^20^, ainda são escassos estudos que descreveram especificamente o perfil sociodemográfico desses profissionais, em âmbito nacional ou regional, o que limitou comparações. Pesquisa realizada na Paraíba com médicos vinculados ao programa entre 2015 e 2016 identificou predominância de profissionais com até cinco anos de formados^21^.

Este estudo apontou uma mudança em relação ao país de nascimento dos profissionais participantes do PMMB, quando comparados aos primeiros ciclos entre 2013-2014. Em 2024, mais de 80,0% dos médicos ativos no programa eram brasileiros, enquanto em julho de 2014, 79,0% dos profissionais eram cubanos e foram recrutados e alocados nos serviços de APS por meio da cooperação internacional com o governo de Cuba e a Organização Pan-Americana de Saúde^22^.

O aumento da proporção de médicos brasileiros, especialmente daqueles formados no Brasil, na composição da força de trabalho do PMMB pode ser atribuído, em parte, à expansão dos cursos de medicina nas últimas décadas e especialmente após a criação do PMM em 2013. Evidências apontaram que entre janeiro de 2014 e fevereiro de 2022 foram abertos 135 novos cursos de medicina, dos quais 68 foram em decorrência do PMM, com maioria de cursos privados, especialmente nas regiões Nordeste, Sudeste e Sul, e localizados em áreas com maior disponibilidade de médicos^23^. Porém, ao considerar apenas a implantação de novos cursos de medicina em universidades federais brasileiras, foi documentada a criação de 30 novos cursos entre 2013 e 2015, todos eles situados em municípios localizados fora dos grandes centros urbanos e das capitais, distribuídos nas cinco macrorregiões brasileiras^24^. Além disso, a expansão recente das vagas de residência médica, especialmente em estados do Nordeste, também sinaliza um processo de descentralização e de interiorização da formação médica, potencialmente associado à maior fixação de profissionais nas regiões onde se especializam^2^.

Apesar do aumento de médicos brasileiros no PMMB, a elevada proporção de intercambistas ainda impõe desafios à fixação profissional e à construção de vínculos duradouros na APS. A atuação de médicos formados no exterior com registros temporários e exclusivos para o programa é mais suscetível a variações na agenda política, como ocorreu em 2018, com a saída dos médicos cubanos após a eleição do ex-presidente Jair Bolsonaro^25^. A descontinuidade abrupta desses profissionais pode comprometer a continuidade do cuidado e a qualidade dos serviços de saúde, pois está associada à redução da oferta de serviços de APS e maior utilização de serviços de urgência e emergência, com impactos negativos nos desfechos em saúde^26^.

Os intercambistas, majoritariamente do sexo masculino e em faixas etárias mais elevadas, estavam predominantemente inseridos em territórios com históricas dificuldades de provimento e de fixação de médicos e localizados em áreas remotas. Esses achados reforçaram que, apesar dos avanços no provimento de médicos na APS desde a criação do PMM em 2013, a alocação de profissionais formados no Brasil em territórios mais vulneráveis ainda é desafiadora. Estratégias para atrair médicos formados no exterior, brasileiros ou estrangeiros, permanecem relevantes para garantir sua presença na atenção primária no país.

Os resultados também mostraram maior frequência de intercambistas em estabelecimentos que atendem populações quilombolas e ribeirinhas. Esse achado corroborou literatura que apontou o PMMB como política efetiva de provimento para populações historicamente desassistidas, com ampliação do acesso à atenção básica em comunidades vulneráveis^5^. Evidências indicaram que médicos estrangeiros, especialmente cubanos, apresentaram maior adaptação a esses contextos em função de terem uma formação orientada à prevenção, à humanização do cuidado e à experiência em missões internacionais, o que favorece uma prática médica alinhada às necessidades de áreas remotas^27^^,^^28^. Essa maior presença também deve ser analisada, contudo, à luz de fatores estruturais associados à baixa fixação de médicos brasileiros em áreas historicamente desassistidas, como remuneração menos atrativa, condições precárias de trabalho e dificuldades de socialização^4^. A inserção desses profissionais em territórios de difícil acesso reforça o papel do programa na redução das desigualdades sociais e territoriais em saúde e evidencia a limitação do mercado interno em suprir essas áreas sem o aporte dos intercambistas.

Aspecto que merece destaque neste estudo é o novo padrão observado entre os intercambistas, predominantemente de brasileiros. Esse achado relativizou a associação imediata entre tipo de registro e país de nascimento, o que revelou que o PMMB congrega trajetórias formativas diversas. Muitos brasileiros buscaram a graduação em outros países diante da alta competitividade no ingresso em cursos de medicina no Brasil, sobretudo antes da recente expansão das escolas médicas nacionais^18^. Ao retornarem, esses profissionais encontram no PMMB uma via legal para inserção profissional no SUS, no âmbito da atuação em serviços de APS, sem enfrentar as exigências regulatórias relacionadas à revalidação do diploma^3^^,^^4^. 

Destacou-se, nesse contexto, a importância do Módulo de Acolhimento e Avaliação, obrigatório para médicos intercambistas. O módulo busca assegurar qualificação mínima para atuação no SUS, ao introduzir esses profissionais às diretrizes da APS, às especificidades epidemiológicas do país e às particularidades socioculturais das comunidades onde atuarão. Para brasileiros formados no exterior, o Módulo de Acolhimento e Avaliação representa não apenas requisito formal, mas oportunidade de atualização e de alinhamento à prática no SUS. O módulo cumpre, assim, dupla função: responde às críticas sobre a inserção de profissionais formados fora do país sem comprovação imediata de competências e atua como dispositivo pedagógico de integração e de regulação, o que contribui para a efetividade do programa em territórios de maior vulnerabilidade social e sanitária^13^.

É interessante notar ainda que foram observadas maiores prevalências de intercambistas em todas as regiões brasileiras, exceto a região Nordeste. Apesar de essa região conter muitos municípios em situação de vulnerabilidade, a expansão e a interiorização do ensino médico, especialmente em universidades federais, pode explicar a menor dependência de médicos formados fora do país^24^. Além disso, é na região Nordeste que se concentram aproximadamente 50,0% de todos os médicos do PMpB, o que pode explicar a menor dependência aos médicos intercambistas para o provimento de vagas nos serviços de APS^29^.

Este estudo foi um dos primeiros de abrangência nacional a descrever o perfil sociodemográfico e os fatores associados ao tipo de registro profissional de médicos ativos no PMMB e evidenciou que a força de trabalho do programa é predominantemente composta por médicos brasileiros, embora haja participação expressiva de intercambistas, especialmente em territórios social e sanitariamente vulneráveis.

A associação entre o tipo de registro profissional, as características individuais dos médicos e as condições estruturais e sociais dos territórios evidenciou padrões distintos de inserção na APS. Esses achados reforçaram a relevância de considerar essas dimensões no planejamento e na avaliação de políticas de provimento, bem como a importância de iniciativas de formação, supervisão e educação continuada para qualificar a atuação desses profissionais e favorecer a permanência deles nos contextos de maior vulnerabilidade.

## Data Availability

O conjunto de dados que dá suporte aos resultados deste estudo não está disponível publicamente.
